# Environmental enrichment for reptiles in European zoos: Current status and perspectives

**DOI:** 10.1017/awf.2023.43

**Published:** 2023-07-03

**Authors:** Alicia Bartolomé, Pau Carazo, Enrique Font

**Affiliations:** Ethology lab, Cavanilles Institute of Biodiversity and Evolutionary Biology, University of Valencia, Spain

**Keywords:** Animal welfare, environmental enrichment, reptiles, survey, taxonomic bias, zoos

## Abstract

Zoos and aquaria are paying increasing attention to environmental enrichment, which has proven an effective tool for the improvement of animal welfare. However, several ongoing issues have hampered progress in environmental enrichment research. Foremost among these is the taxonomic bias, which hinders our understanding of the value of enrichment for neglected groups, such as reptiles. In this study, we evaluated the status of environmental enrichment for reptiles in European zoos using a survey approach. A total of 121 zoos (32% response rate) completed our main survey, focusing on the use of different enrichment types for reptiles. We found significant differences in the use and/or type of enrichment between reptile groups. Tortoises (family Testudinidae) and monitor lizards (genus *Varanus*) were the most enriched taxa while venomous snakes were the least. The enrichment types most used across taxa were structural/habitat design and dietary. A second, more detailed, questionnaire followed, where participants were questioned about specific enrichment techniques. A total of 42 enrichment methods were reported, with two being represented across all taxa: increasing structural/thermal complexity and enrichment objects. Finally, we present information from participating zoos on enrichment goals, assessment methods, sources of information for enrichment ideas, and whether enrichment for reptiles is considered essential and/or implemented routinely. Results suggest that, although usage is widespread across European zoos, our understanding of enrichment for reptiles needs to be re-evaluated, since many of the techniques reported tread a fine line between basic husbandry and actual enrichment.

## Introduction

Most definitions of environmental enrichment centre on the idea of implementing changes in a captive animal’s environment to improve its welfare. As such, environmental enrichment has the potential to be an effective welfare tool, widely and increasingly used in the management of captive animals in a variety of contexts, from private ownership/collections to zoos. For zoos and aquaria especially, increased public demand, and stricter legal and industry regulations are ensuring standards are sought to be improved (Whitham & Wielebnowski 2013; Kagan *et al.* 2015). This has occurred in conjunction with an intensification of zoo-based welfare research and an increased use of evidence-based welfare methods (Mellor *et al.* 2015; Ward *et al.* 2018; Rose *et al.* 2019; Whittaker *et al.* 2021). Furthermore, since its popularisation in the 1990s (Shepherdson 2003), the theoretical framework for environmental enrichment has developed considerably (Newberry 1995; Mellen & MacPhee 2001; Shepherdson 2003; Tarou & Bashaw 2007; Watters 2009; Alligood & Leighty 2015), leading to a steady increase in research studies (Hoy *et al.* 2010). However, in spite of these advances, the use of enrichment as a welfare tool has been impeded by various persistent gaps in knowledge, particularly regarding zoo animal welfare (Melfi 2009).

First, animal welfare science has traditionally focused on avoiding poor welfare (e.g. pain, fear), hence the prevalence of negative indicators and the relative lack of knowledge regarding positive measurements (Yeates & Main 2008; Melfi 2009; Maple & Perdue 2013). In addition, animal welfare in zoos has mostly been assessed using resource-based measurements, namely what resources are provided to the animals (e.g. enclosure dimensions, shelter, nutrition), instead of directly measuring the animals’ physical, physiological, psychological, and behavioural state (i.e. animal-based; European Food Safety Authority [EFSA] 2012). Nevertheless, a shift in perspective is taking place on both fronts (Butterworth *et al.* 2011; Whitham & Wielebnowski 2013). There is increased awareness of the need to use more positive measures of welfare (e.g. Boissy *et al.* 2007; Mellor 2016; Yeates 2016; Williams *et al.* 2018; Yon *et al.* 2019), and to move progressively towards the use of animal-based welfare measures to complement and validate resource-based measurements (e.g. Hewitt & Small 2021; Whittaker *et al.* 2021; Augustine *et al.* 2022; Howard & Freeman 2022).

A second, long-standing issue is the use of tradition, myths, or anecdotal evidence as a staple source of information for husbandry practices (Melfi *et al.* 2005; Melfi 2009; Mendyk 2018; Riley & Rose 2020; Mendyk & Warwick 2023), rather than systematic empirical studies that evaluate the effectiveness of specific welfare tools. Arbuckle (2010, 2013) introduced the term ‘folklore husbandry’ in the context of exotic animal husbandry (particularly herpetofauna) to refer to established methods in husbandry that lack empirical evaluation. Many such methods are shared through dubious or non-peer reviewed literature, such as reports, internet articles, care-sheets and other grey literature (Melfi 2009; Riley & Rose 2020; Tuite *et al.* 2022; Mendyk & Warwick 2023).

Third, environmental enrichment pursues the improvement of an animal’s well-being, so its effectiveness needs to be evaluated empirically. This necessary step is frequently overlooked due to a lack of welfare assessment tools and/or resources and information (Therrien *et al.* 2007; Rosier & Langkilde 2011; Warwick *et al.* 2013; Alligood & Leighty 2015; Alligood *et al.* 2017; Benn *et al.* 2019). In addition, enrichment strategies involve environmental modification, the design of which needs to be carefully tailored to each species’ normal behavioural repertoire and life history (Newberry 1995; Mellen & MacPhee 2001; Shepherdson 2003; Kuppert 2013; Greenberg 2023), which is lacking or limited for many captive species (Mellor *et al.* 2015).

Finally, zoo animal welfare research has traditionally shown a marked taxonomic bias towards mammals, with studies involving other taxa (e.g. reptiles) lagging behind (Burghardt *et al.* 1996; Burghardt 2013, 2020; Kuppert 2013; Mehrkam & Dorey 2014; Rose *et al.* 2019). For instance, Rose *et al.* (2019) conducted a systematic review of zoo and aquarium welfare research from 2009 to 2018 and found that 69% of research papers focused on mammals, similar to that reported in other studies (Melfi 2009; Binding *et al.* 2020). In the case of environmental enrichment, de Azevedo et al. (2007) analysed 744 peer-reviewed enrichment papers (from 1985 to 2004) and reported that 90.2% (n = 635) focused on mammals, with reptiles representing a mere 0.57% (n = 4). Similarly, Alligood and Leighty (2015) found that only 7% (n = 7) of articles pertained to reptiles and amphibians in the period from 2002 to 2014 compared to 90% (n = 86) devoted to mammals; as a case-in-point, primate studies quintupled those with reptiles and amphibians. This taxonomic bias persists, despite ample evidence that reptiles can benefit from properly designed enrichment protocols (e.g. Case *et al.* 2005; Burghardt 2013; Londoño *et al.* 2018; Hoehfurtner *et al.* 2021).

Unfortunately, many gaps in our knowledge exist regarding successful enrichment practices for reptiles, and the current status for reptiles in zoos is relatively unknown (e.g. Eagan 2019; Riley & Rose 2020; Tuite *et al.* 2022). In this study, we evaluated reptile enrichment practices in European zoos. Specifically, we used two surveys to address the following questions: (1) To what extent is enrichment being used for reptiles in European zoos? (2) What are the sources of information, assessment methods, and goals of enrichment for reptiles? (3) Which enrichment types are being used more frequently and for which taxa in particular?

## Materials and methods

### Data collection

We used the survey platform Typeform© to create and distribute two surveys to collect information on the use of enrichment for reptiles in European zoos. The surveys were available in English, French, Spanish, Czech, Italian, and German. We made a contact list of 384 zoos, cross-referencing a list of zoos accredited by the European Association of Zoos and Aquaria (EAZA), the list of zoos and aquariums of the world (Fisken 2020), and internet searches for zoos in all European countries. Zoos that stated clearly on their webpage that they had no reptiles were not included in the contact list. We used public contact information (email and website contact forms) to reach the participants and of the 384 zoos we contacted for the first survey (hereafter referred to as main survey), 60% were EAZA-accredited while the remainder had either a different accreditation status or none at all. We created a second survey (hereafter referred to as follow-up survey) to gather information on specific enrichment measures implemented by zoos that had previously responded to the main survey.

Both surveys were prefaced with short introductions stating the rationale of the study and requesting that the survey be completed by a staff member directly involved in the caring for reptiles, as well as a confidentiality and anonymity statement. The survey platform automatically assigned a random code to each participant for use in data analysis. We also provided a definition of environmental enrichment, as well as examples of the different enrichment types established for this study (Table 1). To enable our results to be compared with a previous study of USA zoos (Eagan 2019), we used the same categories for both reptile groups and enrichment types, except for the merging of ‘natural enrichment devices’ and ‘man-made enrichment devices’ into a single category of ‘enrichment objects and devices.’ We included olfactory enrichment in a broader category entitled ‘sensory enrichment’ (Table 1). The following reptile categories were used: (a) non-venomous snakes; (b) venomous snakes; (c) turtles; (d) tortoises; (e) crocodilians; (f) monitor lizards (genus *Varanus*); and (g) non-monitor lizards.

**Table 1.** Categories and specific examples of environmental enrichment for captive reptiles in zoos (adapted from Eagan 2009)

Category	Examples
Dietary	Novel food presentations (fresh, frozen, live, different textures), use of puzzle-boxes, having food hidden or scattered throughout the enclosure, etc
Sensory	– Olfactory –e.g. application of conspecific, predator or prey scents, or novel scents (spices or perfumes) – Visual –e.g. adding mirrors or pictures to the walls of the enclosure, playing videos – Auditory –e.g. playing sounds to mimic the animal’s natural environment or playing music – Tactile –e.g. adding devices that produce any type of tactile stimulation (for example, different textures)
Training / Behaviour Conditioning	Training for standard husbandry and/or veterinary procedures (e.g. weighing, blood drawing), training for public presentations, etc
Enrichment objects and devices	Addition of branches, rocks, hay, man-made items or toys, etc into the animal’s enclosure
Social	Members of the same species housed together to mimic natural social groupings or mixed species groupings that provide complementary behaviours between species, etc
Structural / Habitat Design	Variety of substrates, terrestrial and aquatic environments, elevated platforms, climbing structures, nesting areas, space changes, etc
Other	Any other practices conducted at this facility that are considered to be enrichment

The main survey was endorsed by the Council of the Societas Europaea Herpetologica (SEH), which provided a support letter to be included with the initial contact email. We sent this first email in October 2020. Two reminders were sent to zoos that did not respond initially in December and January 2021. Data collection for the main survey ended in late February 2021, when we started sending out the follow-up survey to the zoos that had answered the main one. Data collection for the follow-up survey lasted two months.

### Statistical methods

We performed G-tests to determine whether our sample was biased by accreditation status (EAZA vs non-EAZA) relative to the population of zoos that were contacted. We also performed G-tests to determine if significant differences existed between (a) reptile groups for each type of enrichment, and (b) enrichment type use within each reptile group. All *P*-values were adjusted following the Holm-Bonferroni correction for multiple testing (Holm 1979). To control for the effect of zoo, we performed a binomial generalised linear mixed model (GLMM) with ‘Provided to taxa’ as the response variable, ‘Enrichment’, ‘Taxon’ and the interaction between the two as fixed factors, and ‘Zoo’ as a random factor. We coded the binary response variable ‘Provided to taxa’ (1, 0) for each enrichment type if the zoo applied it to the taxa included in their collection (1) or not (0). To explore the use of each enrichment type within each taxon we fitted an additional binomial GLMM with the same predictors for each reptile group. We used ANOVA type III to compute *P*-values.

Most questions in the follow-up survey were open-ended, and the answers were analysed using thematic analysis (Braun & Clarke 2006, 2012). After familiarisation with the data, we started coding the responses using a deductive approach in a descriptive way, generating initial codes for all the enrichment techniques described by the respondents. Once these initial codes were identified, we categorised them into different themes, looking for patterns that matched the general categorisation of enrichment types. Finally, we reviewed the themes and codes, searching for redundancy and eliminating or merging codes and/or elevating them to theme category when necessary. All remaining data were analysed using descriptive statistics.

## Results

### Participants

The main survey had a 32% response rate (n = 121). Among the zoos we contacted initially (n = 384), 229 were accredited by EAZA while 155 were not. Of those zoos that responded to the survey, 73% were accredited by EAZA, 12% had a different accreditation status (e.g. BIAZA, AIZA, SAZA, EPP), and 15% had none. The response rate was higher from EAZA-accredited zoos, with 88 participants (40% of the 229 initially contacted) being accredited by EAZA. In contrast, the response rate for non-EAZA-accredited zoos was 20% (33 out of 155 initially contacted). We found a significant difference in response rates based on accreditation status (G-test: G = 8.58, df = 1; *P* = 0.0034).

Almost all respondents (98%; 118) reported using some form of enrichment for at least some of their animals; out of these 118 zoos, 14% (16) did not use any enrichment for their reptiles. Of the zoos that reported not using enrichment with their reptiles, 14 were EAZA-accredited, one had another accreditation status, and one had no accreditation.

Regarding the person completing the survey on behalf of the zoo, 36% (44) were zookeepers, 31% (37) listed ‘management’ as their occupation, 12% (15) were veterinarians, and 21% (25) listed ‘other’ as their job. Twenty of the participants that chose the latter option (17% of the total) listed ‘curator’ as their occupation using the space provided. The remaining five participants that chose ‘other’ (4% of the total) did not specify their occupation. As ‘curator’ was not listed in the available options, we note that responses under the option ‘management’ may include curatorial roles. Almost half of respondents (45%; 55) had received formal training in environmental enrichment, while 54% (65) had not.

The most common taxa in the respondents’ institutions were tortoises (88%), non-monitor lizards (84%), and non-venomous snakes (83%), followed by turtles (75%) and crocodilians (60%). Monitor lizards were kept in 40% of participants’ zoos and only 22% had venomous snakes.

The remaining demographic information regarding institution type, number of species and number of reptile specimens in the institution, and country where the institution is located can be found in the Supplementary material (Tables S2–S5).

The follow-up survey was sent to the 102 zoos that answered the main survey and provided their reptiles with enrichment. This had a 42% response rate (n = 43).

### Main survey results

**Figure 1.**
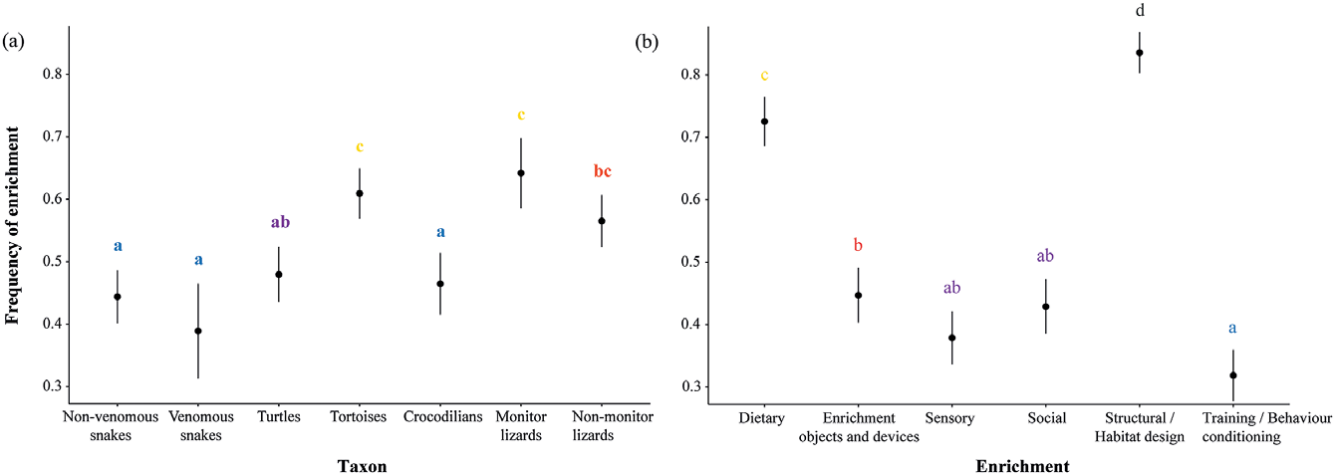
Frequency of general enrichment use for (a) each reptile taxon and (b) each enrichment category utilisation. Letters represent a compact letter display (cld) of every *post hoc* pair-wise comparison. Means not sharing any letter are significantly different by the Tukey-test at the 5% level of significance.

**Figure 2.**
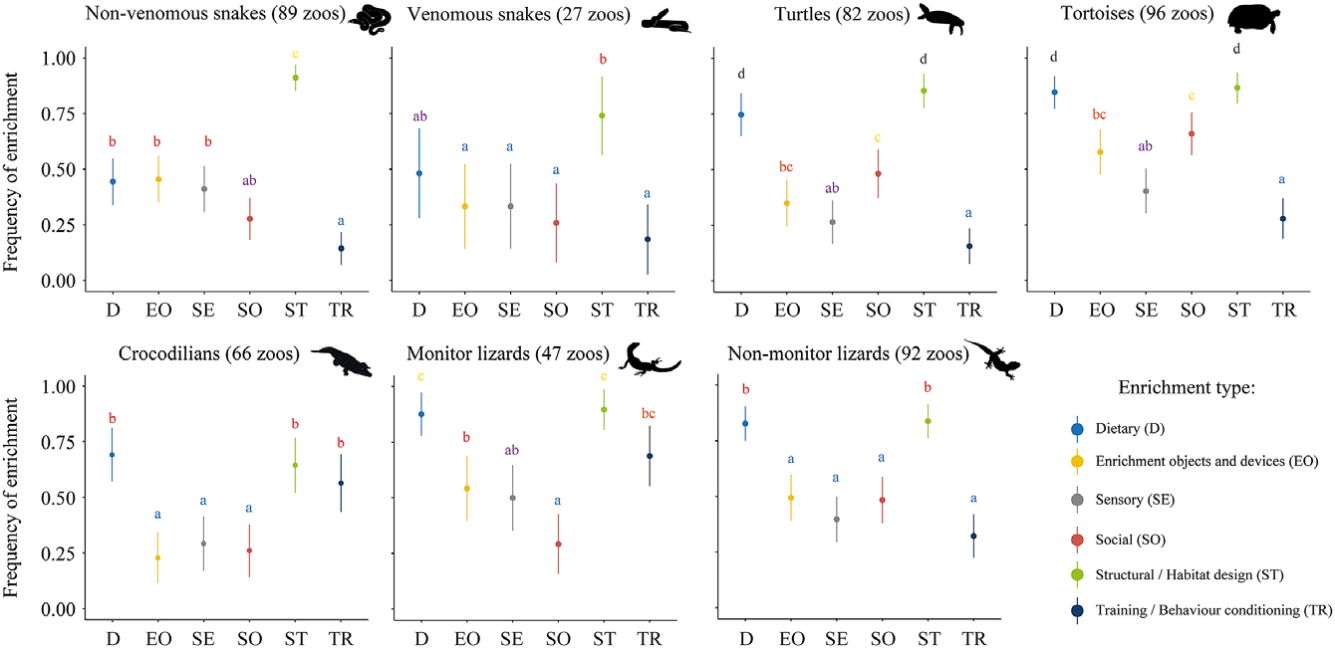
Frequency of each type of enrichment for each reptile taxon. Letters represent a compact letter display (cld) of every *post hoc* pair-wise comparison. Means not sharing any letter are significantly different by the Tukey-test at the 5% level of significance.

**Figure 3.**
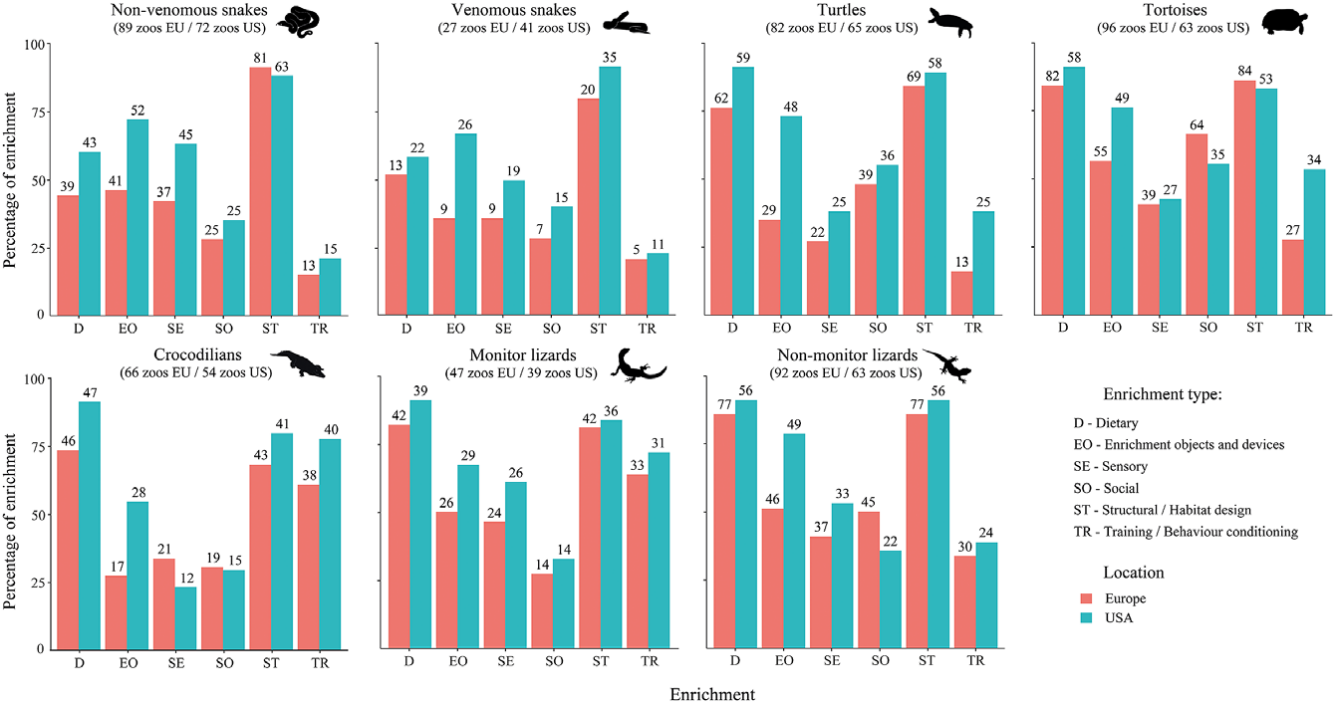
Percentage usage of each type of enrichment for all reptile taxa in European zoos (pink) and USA zoos (blue; Eagan 2019). Numbers in parentheses below each reptile group name represent the total number of zoos providing reptiles with any type of enrichment and having that particular group in their installations. The numbers on top of each bar indicate the total number of zoos providing that specific type of enrichment for the corresponding reptile group. In our study, we used the category of ‘enrichment objects and devices’ as the sole category, unlike the two categories used in the USA study to refer to object enrichment. To enable comparison between both locations, we calculated the mean percentage and number of respondents for the categories ‘natural enrichment devices’ and ‘man-made enrichment devices’ in the USA. As each zoo hosting any reptile group may use different types of enrichment simultaneously, the percentages for a single taxon may exceed 100.

We found significant differences in the type of enrichment across and within reptile groups (Table 2). Additionally, we found a significant interaction between enrichment type and taxon (GLMM [binomial]: *χ*^2^ = 169.19, df = 30; *P* < 0.001), evidencing that the use of different enrichment types varied depending on the taxa. Overall, monitor lizards and tortoises were the most enriched, followed by non-monitor lizards and turtles. Crocodilians, non-venomous snakes and venomous snakes were the least enriched groups (Figure 1). ‘Structural/habitat design’ and ‘dietary’ enrichment stood out as the most commonly used enrichment types (Figure 1). When broken down by reptile group, these two enrichment types remained the most frequently used except for snakes, for whom ‘dietary’ enrichment was provided in a relative lower proportion than for other taxa (Figure 2). ‘Training/behavioural conditioning’ was used the least except for crocodilians and monitor lizards, for whom it was the third most frequent enrichment type (Figure 2). Frequency for all enrichment types was very similar for both groups of snakes but, overall, non-venomous snakes were more likely to be provided with enrichment than venomous ones (Figure 2). Turtles and particularly tortoises stood out in the use of ‘social’ enrichment in comparison with other taxa (Figure 2). Use of enrichment for these taxa in USA zoos (Eagan 2019) follows a similar trend as in Europe (Figure 3).

**Table 2.** Provision of different types of environmental enrichment for reptiles in study zoos (%) and G-test results^1^

	Dietary	Sensory	Training / behaviour conditioning	Enrichment objects and devices	Social	Structural / habitat design	G-test results: Differences within reptile groups
Non-venomous snakes (89)	44% (39)	42 % (37)	15% (13)	46% (41)	28% (25)	**91%** (81)	G: 83.06 *P* < 0.001
Venomous snakes (27)	48% (13)	33% (9)	19% (5)	33% (9)	26% (7)	**74%** (20)	G: 17.82 *P* = 0.003
Turtles (82)	**76%** (62)	27% (22)	16% (13)	35% (29)	48% (39)	**84%** (69)	G: 84.38 *P* < 0.001
Tortoises (96)	**85%** (82)	41% (39)	28% (27)	**57%** (55)	**67%** (64)	**87%** (84)	G: 76.15 *P* < 0.001
Crocodilians (66)	**70%** (46)	32% (21)	**58%** (38)	26% (17)	29% (19)	**65%** (43)	G: 43.54 *P* < 0.001
Monitor lizards (47)	**90%** (42)	51% (24)	**70%** (33)	**55%** (26)	30% (14)	**89%** (42)	G: 36.09 *P* < 0.001
Non-monitor lizards (92)	**84%** (77)	40% (37)	33% (30)	50% (46)	49% (45)	**84%** (77)	G: 63.90 *P* < 0.001
G-test results: Differences between reptile groups	G: 89.82 *P* < 0.001	G: 37.96 *P* < 0.001	G: 52.06 *P* < 0.001	G: 63.45 *P* < 0.001	G: 88.32 *P* < 0.001	G: 85.06 *P* < 0.001	

1 Numbers in parentheses next to each reptile group represent the total number of zoos that are providing reptiles with enrichment and have that particular reptile group in their installations. The total number of zoos that provided reptiles with enrichment was n = 102. Numbers in parentheses under each form of enrichment are the raw number of respondents. *P*-values reported remained significant after applying Holm’s (1979) sequential Bonferroni correction for experiment-wise error rate due to multiple testing. Percentages >50 are in bold.

Participants were questioned about whether they kept track of enrichment provision frequency and type of enrichment used, with 79% declaring that they did not follow any kind of schedule, compared to 21% who did. Of those using a schedule, 90% kept track of how often enrichment was provided and 88% kept track of the type of enrichment provided.

Most respondents (92%) considered that promoting natural/species-specific behaviours was the main reason for enrichment, followed by the facilitation of husbandry/veterinary procedures (50%). Almost half of the participants also chose the reduction of abnormal/stereotyped behaviour as a reason, but it was only considered a primary reason in 1% of cases (Table 3). Responses for ‘other reasons’ included increase of activity levels of reptiles for public viewing, and mental stimulation.

**Table 3.** Reasons given by study zoos for providing reptiles with enrichment^1^

Reasons	*Percentage*	Primary reason	*Percentage*
Promote natural/species-specific behaviours	92.2%	Promote natural/species specific behaviours	74.5%
To facilitate husbandry/veterinary procedures	50%	To facilitate husbandry/veterinary procedures	17.6%
Reduce abnormal/stereotyped behaviour	48%	Public education programme	2.9%
Public education programme	35.3%	Aesthetics	2.9%
Aesthetics	33.3%	Reduce abnormal/stereotyped behaviour	1%
Other	2.9%	Other	1%

1 Percentages calculated out of total of zoos that provide enrichment to reptiles (n = 102). The question for ‘reasons’ was multiple choice but for ‘primary reason’ participants could select only one answer.

Zoos that reported not using enrichment were directed to a set of questions asking the reasons why (Table 4). The main reason was ‘not knowing what to use’ (37%) followed by lack of time (26%) and staff (16%). Lack of money was also a primary reason (11%). Of the 19 zoos that did not provide enrichment to any of their animals, ten had not considered it, according to the respondents.

**Table 4.** Reasons given by study zoos to explain a lack of provision of enrichment^1^

Primary reason	Percentage	Secondary reason	Percentage
Not knowing what to use	37%	Not knowing what to use	47%
Time	26%	Time	32%
Staff	16%	Staff	16%
Money	11%	Money	0%
Other	11%	Other	0%

1 Percentages calculated out of total of zoos that did not provide enrichment to reptiles or to any of their animals (n = 19)

Regarding how enrichment for reptiles was assessed for effectiveness, most participants reported using behavioural measures (i.e. 70% measured whether there was an increase in normal behaviour, while 54% measured use of enrichment by the animals) followed by biological measurements such as growth, weight and shedding (42%). Nineteen percent of respondents did not use any assessment (Table 5).

**Table 5.** Assessment methods utilised by study zoos to ascertain effectiveness of reptile enrichment^1^

Assessment method	Percentage
Increase in normal and/or natural behaviour(s)	70%
Use of enrichment provided	54%
Other biological measurements (e.g. growth, weight, shedding)	42%
Reduction of abnormal and/or unnatural behaviour(s)	40%
Not assessed	19%
Corticosterone levels are analysed	5%
Other physiological stress indicators are analysed (e.g. catecholamine levels, blood glucose levels, heart and blood pressure)	2%
Other	0%

1 Percentages calculated out of total of zoos that provide enrichment to reptiles (n = 102)

Most participants relied on word-of-mouth information from other zookeepers for enrichment ideas for reptiles; along with the internet, this made up for 57% of primary enrichment sources. Other primary sources such as peer-reviewed journal articles and books on environmental enrichment, although reported to be commonly used, were not part of the most used primary sources (Table 6).

**Table 6.** Sources of information for study zoos as regards ideas for reptile enrichment^1^

Source	Percentage	Primary Source	Percentage
Zookeeper information (word-of-mouth)	90%	Zookeeper information (word-of-mouth)	36%
Internet articles	78%	Internet articles	21%
Peer-reviewed journal articles	64%	Zoo accreditation supplied networking	12%
Books on environmental enrichment	58%	Herpetological magazines	10%
Herpetological magazines	54%	Peer-reviewed journal articles	9%
Zoo accreditation supplied networking	40%	Books on environmental enrichment	4%
Other	2%	Other	8%
N/A	1%	N/A	1%

1 Percentages calculated out of total of zoos that provide enrichment to reptiles (n = 102)

### Follow-up survey results

Most of the follow-up survey questions were open-ended, allowing participants to answer freely what they considered to be enrichment in each of the previously established categories and for each reptile group. All answers are presented in Table 7, resulting in 42 codes and 15 themes. Frequencies were calculated using the codified answers within each taxon (see Table S1 in Supplementary material). Due to the large amount of data, Table S1 only reflects the most frequent themes/codes, amounting to ≥ 65% of the total frequency of enrichment techniques for each reptile group.

**Table 7.** General codes (themes) and specific codes for the thematic analysis of the follow-up study’s open-ended questions regarding specific enrichment techniques

General code	Code	Description
Associative learning	Behavioural conditioning for eating/cleaning	Use of vocal and visual cues to feed the animals in a specific part of the enclosure or to move them while cleaning. Name calling (crocodilians and monitor lizards)
	Behavioural conditioning for medical procedures	Training the animal to step on a scale, to draw blood, to enter a box/crate. Use of visual cues to approach veterinarians
	Clicker training	Use of sound (usually a clicking sound) as a conditioned stimulus for training. Positive reinforcement
	Target training	Use of target training for medical reasons and feeding. Positive reinforcement
Cohabit	Cohabit	Unspecified cohabitation of animals
	Heterospecific cohabit	Cohabitation of individuals of different species
	Conspecific cohabit	Cohabitation of individuals of the same species
Varying feeding presentation/patterns	Varying food presentation	Use of tweezers or other unspecified ways to feed the animals
	Varying feeding patterns	Changes in frequency and feeding time
	Frozen food	Providing the animals with frozen food or food inside frozen containers
	Hanging food	Hanging food around the enclosure
	Hiding food	Hiding food around the enclosure
	Food chase	Attaching the prey/food to a stick and moving it around the enclosure to encourage animals to move and chase
	Scatter-feeding	Spreading food around the enclosure
Structural/Thermal complexity	Structural complexity	Floating surfaces, different depths in pool, different zones/habitats, hiding places, elevated platforms
	Climbing structures	Trunks, branches or other structures that are specifically destined for the animal to climb
	Different substrates	Deep substrate, various types of substrates, changing type of substrate and textures
	Nesting area	Nesting area/providing substrates deep enough to allow the construction of nest (for crocodiles)
	Terrestrial and aquatic environments	Availability of both land and water in the same enclosure
	Mimicking natural conditions	Changes in temperature and light following the natural cycle, inducing brumation
	Thermal gradient	Availability of different temperatures along the terrarium, including a hot spot
	Mimicking natural habitat	Natural enclosure design (species-specific)
Enrichment objects	Man-made objects	Boxes, tubes/pipes, paper nozzles, household items, peg boards, tunnels, plastic plants
	Novel items	Unspecified novel objects
	Shell-rubbing object	Brushes for tortoises to rub their shells
	Toys	Coloured balls, ‘kongs’, floating objects (balls, melons…), ‘boomer balls’, mesh balls
	Natural objects	Branches, rocks, leaves, trunks, floating logs, live plants
Olfactory enrichment	Scents from other individuals	Sheds from other species, objects taken from other animals’ enclosures, skin, feathers, faeces
	Smell of prey/food	Objects impregnated with the smell of prey or food
	Fragrant substances	Herbs, spices, essential oils, pungent smells (orange peel, coffee, mint, etc)
	Scent trails	Leaving a trail of some type of smell or blood around the enclosure for the animals to explore
	Unknown scents	Unspecified smells
Live prey/plants	Live plants to eat	Plants planted on the soil of the enclosure
	Live prey	Feeding live prey
Nutritional device	Nutritional device	Feeder ball, ‘kongs’, placing food in boxes or tubes, puzzle box/feeder
Visual enrichment	Visual enrichment	Visual access to conspecifics, mirror
Tactile enrichment	Tactile stimulation	Unspecified tactile stimulation
Human contact	Human contact	Desensitisation to human contact/handling, hand feeding, interaction with the public
Different food items	Different food items	Feeding the animals different types of food/prey
Exploring out of enclosure	Exploring out of enclosure	Allowing the animals to roam out of their enclosures
Varying furniture	Varying furniture	Changes in the enclosure’s furniture, rearrangement of furniture, addition of new furniture
Auditory enrichment	Auditory enrichment	Unspecified auditory enrichment

Two of the most common enrichment techniques for all taxa were ‘structural/thermal complexity’ and ‘enrichment objects’, followed by ‘cohabitation’ and ‘varying feeding presentations/patterns’ (the latter were present in all taxa but monitor lizards and crocodilians, respectively). ‘Varying feeding presentations/patterns’ included hiding and hanging food around the enclosure, among other techniques of which ‘food chase’ stood out for non-venomous snakes. Another nutritional enrichment used by most zoos is ‘different food items’, present in all taxa except snakes. ‘Varying furniture’ was an enrichment technique only used for turtles and non-venomous snakes, while ‘olfactory enrichment’ made up an important percentage of enrichment for both groups of snakes and non-monitor lizards. Finally, ‘associative learning’ was only prevalent for crocodilians and monitor lizards, and it encompassed ‘target training’, ‘behavioural conditioning for medical procedures’ and ‘behavioural conditioning for eating/cleaning’ (Table S1).

When asked whether enrichment for reptiles was considered an essential practice or something tackled as a luxury, more than half (60%; 26) reported it to be essential. A smaller percentage (14%; 6) expressed the opinion that for them enrichment was a luxury for reptiles while the rest (26%; 11) considered it to be dependent upon the taxon. Those who chose the latter option were asked for which of their reptiles was enrichment considered essential. All participants that checked this option and had monitor lizards in their collection (9) considered it essential for them. The opposite was seen with venomous snakes: no zoos with venomous snakes (2) considered enrichment essential for them. Regarding the rest of the groups, 56% of zoos deemed enrichment essential for crocodiles (9), 38% for tortoises (8), 33% for turtles (9), 25% for non-monitor lizards (8), and 11% for non-venomous snakes (9).

Open-ended questions were formulated to explore why enrichment was considered essential, a luxury, or group dependent. For those who considered it essential, the reasons were as follows: general well-being (38%), promotion of natural behaviour (31%), mental health (27%), physical health (15%), basic husbandry measure (12%), better management (8%), promotion of reproduction (4%), ethical reasons (4%), avoidance of boredom (4%), decreased stress (4%), and improved quality of life (4%). When asked why enrichment was considered a luxury for reptiles in their installations, the reasons listed, in equal proportion, were: lack of time (33.3%), lack of staff (33.3%), and enrichment considered less important for reptiles than for other groups (33.3%). Responses from participants that answered that the need for enrichment varied according to taxon included: difficulty in implementing, financial restrictions, lack of validation for enrichment in some groups, and insufficient resources to enable enrichment for all species or individuals. To this question, several respondents added information, particularly referring to monitor lizards and crocodilians: “Large, charismatic species and enrichment form part of public engagement” (referring to Komodo dragons [*Varanus komodoensis*]); “I think that monitor lizards and crocodilians are more intelligent than other reptiles”; “There are more studies being made on this group” (referring to monitor lizards); “They have different necessities than the rest” (referring to monitor lizards). We also questioned participants whether enrichment for reptiles was used routinely, or reactive to arising issues (e.g. behavioural, medical problems). More than half of respondents answered that enrichment in their facility was used routinely (56%; 24), while 12% (5) used it to deal with problems (reactive). Thirty-three percent (14) used it routinely for some reptiles but reactively for others. Regarding this last option, 100% of zoos with monitor lizards in their collection used it routinely for them (8), followed by 89% for crocodilians (10), 50% for non-monitor lizards (13), 29% for tortoises (14), 21% for non-venomous snakes (14), 20% for turtles (10) and 0% for venomous snakes.

Finally, we asked participants to rate, on a scale from 1 to 10, how important they considered enrichment for several groups of animals. Mean scores calculated out of the total of respondents (n = 41) were: 9.3 (± 1.4) for mammals, 8.6 (± 1.4) for birds, 7.2 (± 1.9) for reptiles, 5.1 (± 2.8) for fishes, and 4.4 (± 2.6) for invertebrates.

## Discussion

Using a survey approach, we have examined the current status of environmental enrichment for reptiles in European zoos. Our results allow us to gain a picture of the current practices for reptiles in Europe and to compare the situation to that in the USA. Use of environmental enrichment seems widespread in European zoos, particularly dietary and structural enrichment. Our surveys provided data on enrichment type, and specific enrichment techniques used within each category of enrichment. These data are relevant to help in understanding how reptile enrichment is perceived by practitioners. Additionally, in both surveys, we asked an array of complementary questions that provide valuable insight into the limitations affecting enrichment implementation. Despite being generally considered as important, enrichment implementation presents greater challenges in reptiles in terms of understanding and application compared to various other animal groups, possibly as a result of scarcity of information and a number of other limitations. Foremost among these is the taxonomic bias (‘taxonomic chauvinism’; e.g. Bonnet *et al.* 2002; Pawar 2003) that affects reptiles and other neglected groups.

### Importance of environmental enrichment for reptiles and general use

Our results would suggest that the use of environmental enrichment for reptiles in zoos and aquaria is widespread in Europe, however only 121 zoos out of the 384 originally contacted (32% response rate) responded. Also, a greater representation of EAZA-accredited zoos among survey respondents was noted, with a response rate double that of non-accredited zoos or zoos with different accreditation status. It is possible therefore that sampling bias has overestimated the proportion of zoos conducting environmental enrichment with reptiles. Almost all our respondents (118; 98%) used environmental enrichment in their facilities but 14% (16) used it for other animals but not for reptiles. Similarly, all zoos (76) in the USA study (Eagan 2019) reported using enrichment for their animals, but only 5% (4) provided enrichment to some of their animals but not to reptiles. The latter may only provide enrichment for certain token species or to most animals in their care except reptiles (and all the possibilities in-between). When asked about the rationale for allocation of environmental enrichment, zoos that did not enrich their animals reported time, staff and budget constraints, echoing the limitations already detected in other studies (Hoy *et al.* 2010; Riley & Rose 2020; Tuite *et al.* 2022). However, the main reason for the absence of enrichment reported in our study was a lack of knowledge regarding what is appropriate to use.

The taxonomic bias affecting research on environmental enrichment plays a major role in this lack of information (de Azevedo et al. 2007; Melfi 2009; Rose *et al.* 2019; Riley & Rose 2020). The EAZA issues Best Practice Guidelines for species or groups of closely related species kept in zoos (EAZA 2022). Only six of the Guidelines available (as per January 2023) concern reptile species, while mammals garner 32 documents (the rest include one report for fish, four for invertebrates, seven for amphibians, and ten for birds). This example illustrates how taxonomic bias affects the amount of information readily available for zoos. Also, most enrichment practices in non-mammalian species are modelled on enrichment practices designed for mammals, which can be problematic when applied without proper modification and/or evaluation (Mendyk & Augustine 2023). At its inception, environmental enrichment was designed as a means of promoting activity and countering behavioural or welfare problems (a reactive, rather than proactive, approach) in mammals, mainly primates (Young 2003). Later, its study progressed to a behavioural engineering approach in which the primary stimulus utilised was food (Young 2003; Fernandez & Martin 2021). Exercise and increased activity levels also became important focal points of research (mostly in primates and felines), which shaped the subsequent development of environmental enrichment science and its application. When translated to ectotherms, this inertia is inadequate given that they do not need such frequent feeding, have lower metabolic rates, and their activity is more dependent on ambient temperature.

Zoo professionals also report other barriers to enrichment. For instance, there is a perceived lack of institutional support (i.e. limited interest from zoo management, scarcity of resources, money, or staff) and lack of interest from the wider community in the use of enrichment (Riley & Rose 2020), and a prioritisation of health and physical well-being over psychological or mental well-being (Tuite *et al.* 2022). These barriers hamper the widespread use of enrichment; therefore, it is worth considering how enrichment is prioritised for different taxa.

In our follow-up survey we asked participants to rate, on a scale from one to ten, the importance of enrichment for different taxa: reptiles scored third after mammals and birds, which again reflects the taxonomic bias affecting animal welfare and enrichment science. Many reasons contribute to reptiles lagging behind in any prioritisation enrichment plan. Underlying those reasons is the perception that reptiles are perceptually/cognitively limited animals whose requirements in captivity are modest compared to other groups, and are capable of tolerating even the most impoverished captive conditions (Burghardt 2013; Maple & Perdue 2013). These misconceptions, which often stem from inadequate or misleading information, have been sternly criticised (e.g. Burghardt 1977; Warwick *et al.* 1994; Burghardt 1997; Young 2003; Font *et al.* 2023; Mendyk & Warwick 2023), but these have had limited success in correcting such widespread misconceptions about reptile biology and behaviour. Recently, public interest in animal welfare has prompted a perspective shift in how reptiles are perceived and treated. Studies on reptile sentience (e.g. for reviews, see Lambert *et al.* 2019; Learmonth 2020), cognition (e.g. Cooper *et al.* 2019; Font 2019, 2020; Burghardt 2020; LaDage *et al.* 2012; Szabo *et al.* 2021), play (e.g. Burghardt 2005, 2013, 2015; Dinets 2015; Kane *et al.* 2019), and complex sociality (e.g. Doody *et al.* 2013, 2021; Gardner *et al.* 2016; Dinets 2017; Skinner & Miller 2020; Baker *et al.* 2023) have accumulated in recent years. Although this shift in perception is positive, the lack of scientific validation for reptile husbandry practices is a persistent problem, particularly important given the large number and diversity of reptiles being traded into captivity (e.g. Warwick 2014; Draper & Jones 2017; Warwick *et al.* 2018; Altherr & Lameter 2020).

### Foundations of enrichment for reptiles in European zoos

The use of reliable, validated welfare tools is of utmost importance in the pursuit of successful husbandry practices (Alligood & Leighty 2015; Benn *et al.* 2019). This process requires a solid basis, from the conception of any husbandry idea to the evaluation of its effectiveness; therefore, participants were questioned on the foundations of current zoo enrichment practices for reptiles. In both Eagan’s (2019) and the present study, the primary source of enrichment ideas was word-of-mouth, i.e. information from other zookeepers and professionals. Peer-reviewed articles are used as a primary source behind internet articles and information provided by zoos’ accreditation institutions. Riley and Rose (2020) reported low use of journal articles by zoo practitioners, and their perception was that literature availability was limited when making decisions on enrichment. It is possible that the scientific advances on reptile welfare and enrichment go unnoticed either because they are published outside the direct scope of zoological publishing (Mendyk 2022), or because accessibility to peer-reviewed literature is limited (Riley & Rose 2020), so zoo professionals rely on more proximate or familiar sources. Some of those alternative sources fall into the realm of grey literature, and this can be problematic due to a lack of validation. Although easily disseminated and more accessible, information distributed through non-reviewed sources, such as internet articles or word-of-mouth, has a higher probability of containing misinformation (Warwick *et al.* 2013; Warwick 2014; Draper & Jones 2017; D’Cruze *et al.* 2020; Mendyk & Warwick 2023). Thus, these types of sources are not only unreliable (Loughman 2020), but can become fertile ground for the perpetuation of folklore husbandry. To move towards evidence-based husbandry, a key distinction has to be made between husbandry practices that are backed by empirical evidence vs ones that are accepted only because of tradition (Arbuckle 2013). Otherwise, there is a risk of perpetuating collective knowledge that is at best ineffective and at worse harmful for reptiles in captivity. For instance, one of the most patent examples of folklore husbandry relates to snakes and the size of their enclosures. The use of racks for snake-keeping is commonplace (Loughman 2020), and typically feature small boxes that prevent snakes fully stretching (Warwick *et al.* 2019). Some of the reasons why snake enclosures are often small and simple derive from folklore (e.g. snakes are sedentary animals that dislike open or large spaces; see Warwick *et al.* 2019), and sources that recommend their use are based on outdated practices no longer supported by scientific evidence (Warwick *et al.* 2021).

In the present study, the most frequently used assessment methods for effectiveness were an increase in normal behaviour and use of the enrichment provided, similar to Eagan’s study (2019). In both studies, a similar percentage of zoos reported a lack of formal assessment of their enrichment techniques (14 and 19% in USA and Europe, respectively). Animal-based measurements, although of increasing importance in reptile welfare, lag behind resource-welfare measurements (Benn *et al.* 2019). For example, physiological welfare indicators (e.g. corticosterone levels) have barely been studied in reptiles and need to be validated in a wider range of species (Benn *et al.* 2019; Gangloff & Greenberg 2023). In our study, we only asked about the use of animal-based measurements, so we cannot know whether there is a preference for animal-based measurements in European zoos, or if the use of resource-based measurements is still dominant for reptile species, as has traditionally been the case (Benn *et al.* 2019; Jones et al. 2022). Regardless, behavioural methods seem to be of importance for zoos, probably because they are easier to implement than invasive or resource-intensive methods (Whittaker *et al.* 2021; Jones *et al.* 2022).

When using behavioural indicators of welfare, it is essential they are applied within a species-specific context that will allow proper interpretation (Bacon 2018; Benn *et al.* 2019; Spain *et al.* 2020). For reptiles especially, this comes into direct conflict with the relative scarcity of behavioural observations in the wild (Warwick *et al.* 2013), making it difficult to establish what qualifies as abnormal behaviour in captivity. A key but frequently overlooked issue is that reptiles are a highly diverse group, with close to 12,000 currently recognised species. This adds to the challenge of identifying and validating behavioural welfare indicators (Burghardt & Layne-Colon 2023). Finally, interpreting reptile behaviour poses a special challenge due to its characteristics and to our own anthropomorphic tendencies (e.g. Burghardt 1991; Rivas & Burghardt 2002; Batt 2009; Wilkins *et al.* 2015; Mather 2019). For example, reptilian ectothermic physiology and lack of facial expressions have been proposed as two main factors that may inhibit or complicate assessment of aversive states such as fear or pain (Warwick *et al.* 2017; Whitehead 2018; Williams & Beck 2021), which in many cases can go unnoticed (Malik 2018). Also, there is ample evidence that a species’ likeability decreases with phylogenetic distance and dissimilarity to our species (e.g. Batt 2009; Miralles *et al.* 2019). In general, reptiles tend to be disliked by humans and, particularly in the case of snakes, are target of a myriad of negative cultural beliefs (Ceríaco 2012; Whitehead 2018; Janovcová et al., 2019; Da Silva *et al.* 2021). Yet, not every reptile group is perceived in the same manner.

### Differences in enrichment use for each reptile group and specific enrichment techniques

Several studies indicate that some reptiles, such as turtles and tortoises, are perceived in a positive light in contrast to, for instance, snakes (Czech *et al.* 1998; Janovcová *et al.* 2019; Da Silva *et al.* 2021). This taxonomic bias is also reflected in our results. Tortoises and monitor lizards received the most enrichment amongst all reptile groups, and all respondents that had monitor lizards in their installations considered it essential for them. The number of respondents for these questions was small, but the narrative in open-ended questions provides insight into why enrichment is perceived differently across taxonomic groups. Some specified that the animals that most likely benefit from enrichment are those that are more active and show more appetite, which mirrors the aforementioned historical inertia for mammal environmental enrichment. One of the respondents wrote that “[Enrichment] may benefit other groups just as much, but it is difficult to tell, when no behaviours are displayed as a reaction to the enrichment.” Particularly with monitor lizards, participants invoked their ‘high intelligence’ and ‘high interaction with environment.’ Size and longevity also seem to be important factors when prioritising the use of enrichment within reptiles, particularly in favour of large, long-lived species such as giant tortoises or crocodiles. It should be noted, however, that the use of enrichment for different reptiles may be affected by how easily its application is perceived (i.e. certain types of enrichment may be thought to be more difficult to implement in reptiles that are potentially dangerous for their caretakers, such as crocodiles or venomous snakes). In any case, as noted by previous studies, research and husbandry efforts focus on large, charismatic species that appeal to the public (e.g. Melfi 2009; Carr 2016; Albert *et al.* 2018; Hosey *et al.* 2020) and for which more studies are available. Moreover, scientists and zoo professionals may devote greater efforts to studying more familiar species as there is more information available for them (Rose *et al.* 2019). Lack of understanding as to what constitutes environmental enrichment was an issue for some of our respondents. For instance, one participant commented that “the problem is that enrichment is not anything definite in reptile-keeping”, while another pointed out that “[…] we are just beginning to understand the importance of reptile enrichment in husbandry.”

Enrichment refers to a change in the environment that fulfils some welfare goals and results in an improvement for the animal. It does not refer to any change or modification for which the outcome is unknown, even though that is how the term is sometimes used (Newberry 1995). In this sense, the present study collected a lot of information on what zoo professionals consider enriching for reptiles. In our follow-up survey we focused on retrieving information on specific enrichment techniques. This resulted in a wide diversity of responses and raises the question as to whether some of these reported enriching techniques should indeed be considered enrichment (Mendyk & Augustine 2023). For example, one of the most commonly reported enrichment techniques in five out of seven reptile groups in our study was the use of different food items: can feeding reptiles with multiple types of food/different prey be considered enrichment or is it simply a matter of basic husbandry? Discrepancies in what qualifies as enrichment for reptiles may be another contributing factor to the taxonomic bias (Riley & Rose 2020), particularly when approaching enrichment from a mammalian perspective (Mendyk & Augustine 2023). Perhaps, what some consider enrichment is just controlled deprivation (Burghardt 1996, 2013).

## Animal welfare implications and conclusion

The need for enrichment has long been recognised as an integral part of husbandry; its use seems widespread in European zoos, but there are also many limitations that prevent its full and consistent implementation. All the gaps affecting animal welfare mentioned above (see *Introduction*) likely have a greater impact on taxa that have traditionally been neglected. Although reptile welfare is subject to increasing attention, further research is needed on their ecology, behaviour, husbandry, validation of welfare methods, etc. Our study highlights some of the main barriers limiting the use of environmental enrichment for reptiles (such as a scarcity of validated assessment methods, limited access to literature, or lack of well-grounded ideas), underscoring the urgent need for a shift in perspective in reptile environmental enrichment. For instance, zookeepers acknowledge the absence of a clear understanding of enrichment, which can result in mistaking fundamental basic husbandry for enrichment protocols. Hence, reptile enrichment research would benefit from adopting a broader perspective, such as considering a wider array of enrichment types, increasing the number of species studied, and validating different welfare assessment methods. Consider, for example, the use of chemosensory enrichment, which seems to lag behind other types of enrichment despite the critical importance of chemoreception for reptiles (e.g. Chiszar *et al.* 1995; Bashaw *et al.* 2016; Londoño *et al.* 2018).

Furthermore, our study adds to the growing body of literature that evaluates the relationship between captive reptiles and their human carers with regards to different aspects of reptile welfare. This type of approach allows scientists and professionals from other areas to weave different paths of communication that should contribute to mitigate the disconnect between practice and science (Loughman 2020; Riley & Rose 2020; Mendyk 2022). Our results also reveal a wide set of specific enrichment techniques that are currently being used for reptiles in zoos. In order to clarify the boundary between basic husbandry and enrichment, further research should inquire about what is considered enriching for each species according to zoo professionals.

To conclude, our study highlights the importance of basing species-specific environmental enrichment protocols on currently available information, and to empirically assess them for effectiveness. Zoos are appropriate venues to focus on traditionally disregarded groups such as reptiles, which are also likely to benefit the most from this research.

## Supplementary material

For supplementary material accompanying this paper visit https://doi.org/10.1017/awf.2023.43. click here to view supplementary material

## References

[r1] Albert C, Luque GM and Courchamp F 2018 The twenty most charismatic species. PloS One 13: e0199149. 10.1371/journal.pone.0199149 PMC6037359 29985962

[r2] Alligood C and Leighty K 2015 Putting the “E” in SPIDER: Evolving trends in the evaluation of environmental enrichment efficacy in zoological settings. Animal Behavior and Cognition 2: 200–217. 10.12966/abc.08.01.2015

[r3] Alligood CA, Dorey NR, Mehrkam LR and Leighty KA 2017 Applying behavior‐analytic methodology to the science and practice of environmental enrichment in zoos and aquariums. Zoo Biology 36: 175–185. 10.1002/zoo.21368 29165867

[r4] Altherr S and Lameter K 2020 The rush for the rare: Reptiles and amphibians in the European pet trade. Animals 10: 2085. 10.3390/ani10112085 33182744 PMC7697995

[r5] Arbuckle K 2010 Suitability of day‐old chicks as food for captive snakes. Journal of Animal Physiology and Animal Nutrition 94: e296–e307. 10.1111/j.1439-0396.2010.01011.x 20626504

[r6] Arbuckle K 2013 Folklore husbandry and a philosophical model for the design of captive management regimes. Herpetological Review 44: 448–452.

[r7] Augustine L, Baskir E, Kozlowski CP, Hammack S, Elden J, Wanner MD, Franklin AD and Powell DM 2022 Investigating welfare metrics for snakes at the Saint Louis Zoo. Animals 12: 373. 10.3390/ani12030373 35158696 PMC8833826

[r8] Bacon H 2018 Behaviour-based husbandry: A holistic approach to the management of abnormal repetitive behaviors. Animals 8: 103. 10.3390/ani8070103 29954148 PMC6070902

[r9] Baker CJ, Frère CH, Franklin CE, Campbell HA, Irwin TR and Dwyer RG 2023 Long-term tracking reveals a dynamic crocodylian social system. Animal Behaviour 199: 59–78. 10.1016/j.anbehav.2023.02.015

[r10] Bashaw MJ, Gibson MD, Schowe DM and Kucher AS 2016 Does enrichment improve reptile welfare? Leopard geckos (*Eublepharis macularius*) respond to five types of environmental enrichment. Applied Animal Behaviour Science 184: 150–160. 10.1016/j.applanim.2016.08.003

[r11] Batt S 2009 Human attitudes towards animals in relation to species similarity to humans: A multivariate approach. Bioscience Horizons 2: 180–190. 10.1093/biohorizons/hzp021

[r12] Benn AL, McLelland DJ and Whittaker AL 2019 A review of welfare assessment methods in reptiles, and preliminary application of the Welfare Quality® Protocol to the pygmy blue-tongue skink, *Tiliqua adelaidensis*, using animal-based measures. Animals 9: 27. 10.3390/ani9010027 30658490 PMC6356264

[r13] Binding S, Farmer H, Krusin L and Cronin K 2020 Status of animal welfare research in zoos and aquariums: Where are we, where to next? Journal of Zoo and Aquarium Research 8: 166–174. 10.19227/jzar.v8i3.505

[r14] Boissy A, Manteuffel G, Jensen MB, Moe RO, Spruijt B, Keeling LJ, Winckler C, Forkman B, Dimitrov I, Langbein J, Bakken M, Veissier I and Aubert A 2007 Assessment of positive emotions in animals to improve their welfare. Physiology & Behavior 92: 375–397. 10.1016/j.physbeh.2007.02.003 17428510

[r15] Bonnet X, Shine R and Lourdais O 2002 Taxonomic chauvinism. Trends in Ecology & Evolution 17: 1–3. 10.1016/s0169-5347(01)02381-3

[r16] Braun V and Clarke V 2006 Using thematic analysis in psychology. Qualitative Research in Psychology 3: 77–101. 10.1191/1478088706qp063oa

[r17] Braun V and Clarke V 2012 Thematic analysis. In: Cooper H, Camic PM, Long DL, Panter AT, Rindskopf D and Sher KJ (eds) APA Handbook of Research Methods in Psychology, Volume 2. Research Designs: Quantitative, Qualitative, Neuropsychological, and Biological pp 57–71. American Psychological Association: Washington, DC, USA. 10.1037/13620-004

[r18] Burghardt GM 1977 Learning processes in reptiles. In: Gans C and Tinkle DW (eds) The Biology of the Reptilia, Volume 7. Ecology and Behaviour pp 555–681. Academic Press: London, UK. 10.2307/1443713

[r19] Burghardt GM 1991 Cognitive ethology and critical anthropomorphism: A snake with two heads and hog-nose snakes that play dead. In: Ristau CA (eds) Cognitive Ethology: The Minds of Other Animals pp 53–90. Lawrence Erlbaum Associates: Hillsdale, New Jersey, USA.

[r20] Burghardt GM 1996 Environmental enrichment or controlled deprivation? In: Burghardt GM, Bielitzki JT, Boyce JR and Schaeffer DO (eds) The Well-being of Animals in Zoo and Aquarium Sponsored Research pp 91–101. Scientists Center for Animal Welfare: Greenbelt, MD, USA.

[r21] Burghardt GM 1997 Amending Tinbergen: A fifth aim for ethology. In: Mitchell S, Thompson NS and Miles HL (eds) Anthropomorphism, Anecdotes, and Animals pp 254–276. SUNY Press: Albany, NY, USA.

[r22] Burghardt GM 2005 The Genesis of Animal Play: Testing the Limits. MIT Press: Cambridge, MA, USA. 10.7551/mitpress/3229.001.0001

[r23] Burghardt GM 2013 Environmental enrichment and cognitive complexity in reptiles and amphibians: Concepts, review, and implications for captive populations. Applied Animal Behaviour Science 147: 286–298. 10.1016/j.applanim.2013.04.013

[r24] Burghardt GM 2015 Play in fishes, frogs and reptiles. Current Biology 25: R9–R10. 10.1016/j.cub.2014.10.027 25562306

[r25] Burghardt GM 2020 The learning repertoire of reptiles. In: Melfi, VA, Dorey NR and Ward SJ (eds) Zoo Animal Learning and Training pp 227–280. Wiley-Blackwell: Hoboken, NJ, USA. 10.1002/9781118968543.oth12

[r26] Burghardt GM and Layne-Colon DG 2023 Effects of ontogeny, rearing conditions, and individual differences on behaviour: Welfare, conservation, and invasive species implications. In: Warwick C, Arena PC and Burghardt GM (eds) Health and Welfare of Captive Reptiles, Second Edition pp 287–321. Springer Publishers: London, UK.

[r27] Burghardt GM, Ward B and Rosscoe R 1996 Problem of reptile play: Environmental enrichment and play behavior in a captive Nile soft‐shelled turtle, *Trionyx triunguis*. Zoo Biology: Published in affiliation with the American Zoo and Aquarium Association 15: 223–238. 10.1002/(SICI)1098-2361(1996)15:3<223::AID-ZOO3>3.0.CO;2-D

[r28] Butterworth A, Mench JA, Wielebnowski N and Olsson IAS 2011 Practical strategies to assess (and improve) welfare. In: Appleby MC, Olsson AS and Galindo F (eds) Animal Welfare, Third Edition pp 232–250. CABI: Wallingford, Oxon, UK; Boston, MA, USA. 10.1079/9781845936594.0200

[r29] Carr N 2016 An analysis of zoo visitors’ favourite and least favourite animals. Tourism Management Perspectives 20: 70–76. 10.1016/j.tmp.2016.07.006

[r30] Case BC, Lewbart GA and Doerr PD 2005 The physiological and behavioural impacts of the preference for an enriched environment in the eastern box turtle (*Terrapene carolina carolina*). Applied Animal Behaviour Science 92: 353–365. 10.1016/j.applanim.2004.11.011

[r31] Ceríaco LM 2012 Human attitudes towards herpetofauna: The influence of folklore and negative values on the conservation of amphibians and reptiles in Portugal. Journal of Ethnobiology and Ethnomedicine 8: 1–13. 10.1186/1746-4269-8-8 22316318 PMC3292471

[r32] Chiszar D, Tomlinson WT, Smith HM, Murphy JB and Radcliffe CW 1995 Behavioural consequences of husbandry manipulations: indicators of arousal, quiescence and environmental awareness. In: Warwick C, Frye FL and Murphy JB (eds) Health and Welfare of Captive Reptiles pp 186–204. Chapman & Hall/Kluwer: London, UK; New York, NY, USA.

[r33] Cooper T, Liew A, Andrle G, Cafritz E, Dallas H, Niesen T, Slater E, Stockert J, Vold T, Young M and Mendelson III JM 2019 Latency in problem solving as evidence for learning in varanid and helodermatid lizards, with comments on foraging techniques. Copeia 107: 78–84. 10.1643/ch-18-119

[r34] Czech B, Krausman PR and Borkhataria R 1998 Social construction, political power, and the allocation of benefits to endangered species. Conservation Biology 12: 1103–1112. 10.1046/j.1523-1739.1998.97253.x

[r35] D’Cruze N, Paterson S, Green J, Megson D, Warwick C, Coulthard E, Norrey J, Auliya M and Carder G 2020 Dropping the ball? The welfare of ball pythons traded in the EU and North America. Animals 10: 413. 10.3390/ani10030413 32131452 PMC7143053

[r36] Da Silva MXG, Braga-Pereira F, Da Silva MC, de Oliveira JV, de Faria Lopes S and Alves RRN 2021 What are the factors influencing the aversion of students towards reptiles? Journal of Ethnobiology and Ethnomedicine 17: 1–10. 10.1186/s13002-021-00462-z 34011374 PMC8136183

[r37] De Azevedo CS, Cipreste CF and Young RJ 2007 Environmental enrichment: A GAP analysis. Applied Animal Behaviour Science 102: 329–343. 10.1016/j.applanim.2006.05.034

[r38] Dinets V 2015 Play behavior in crocodilians. Animal Behavior and Cognition 2: 49–55. 10.12966/abc.02.04.2015

[r39] Dinets V 2017 Coordinated hunting by Cuban boas. Animal Behaviour and Cognition 4: 24–29. 10.12966/abc.02.02.2017

[r40] Doody JS, Burghardt GM and Dinets V 2013 Breaking the social-non-social dichotomy: A role for reptiles in vertebrate social behavior research? Ethology 119: 1–9. 10.1111/eth.12047

[r41] Doody JS, Dinets V and Burghardt GM 2021 The Secret Social Lives of Reptiles. Johns Hopkings University Press: Baltimore, MD, USA. 10.1353/book.84105

[r42] Draper C and Jones M 2017 The future of keeping pet reptiles and amphibians. The Veterinary Record 181: 629–630. 10.1136/vr.j5711 29222153

[r43] EFSA Panel on Animal Health and Welfare (AHAW) 2012 Statement on the use of animal‐based measures to assess the welfare of animals. EFSA Journal 10: 2767. 10.2903/j.efsa.2012.2767

[r44] Eagan T 2019 Evaluation of enrichment for reptiles in zoos. Journal of Applied Animal Welfare Science 22: 69–77. 10.1080/10888705.2018.1490182 30052062

[r45] EAZA 2022 *Best Practice Guidelines.* https://www.eaza.net/conservation/programmes/#BPG

[r46] Fernandez EJ and Martin AL 2021 Animal training, environmental enrichment, and animal welfare: a history of behavior analysis in zoos. Journal of Zoological and Botanical Gardens 2: 531–543. 10.31234/osf.io/wv68k

[r47] Fisken FA 2020 Index to list of zoos and aquariums of the World. International Zoo Yearbook 54: 375–392. 10.1111/izy.12258

[r48] Font E 2019 Rapid learning of a spatial memory task in a lacertid lizard (*Podarcis liolepis*). Behavioural Processes 169: 103963. 10.1016/j.beproc.2019.103963 31545992

[r49] Font E 2020 Squamate cognition. In: Vonk J and Shackelford T (eds) Encyclopedia of Animal Cognition and Behavior pp 1–10. Springer: Cham, Switzerland. 10.1007/978-3-319-47829-6_93-1

[r50] Font E, Burghardt GM and Leal M 2023 Reptile brains, behavior, and cognition: Multiple misconceptions. In: Warwick C, Arena, PC and Burghardt GM (eds) Health and Welfare of Captive Reptiles, Second Edition pp 211–238. Springer Publishers: London, UK.

[r51] Gangloff EJ and Greenberg N 2023 Biology of stress. In: Warwick C, Arena, PC and Burghardt GM (eds) Health and Welfare of Captive Reptiles, Second Edition pp 93–142. Springer Publishers: London, UK.

[r52] Gardner MG, Pearson SK, Johnston GR and Schwarz MP 2016 Group living in squamate reptiles: A review of evidence for stable aggregations. Biological Reviews 91: 925–936. 10.1111/brv.12201 26052742

[r53] Greenberg N 2023 Ethologically informed design and DEEP ethology in theory and practice. In: Warwick C, Arena, PC and Burghardt GM (eds) Health and Welfare of Captive Reptiles, Second Edition pp 379–416. Springer Publishers: London, UK.

[r54] Hewitt L and Small A 2021 Welfare of farmed crocodilians: Identification of potential animal-based measures using elicitation of expert opinion. Animals 11: 3450. 10.20944/preprints202111.0196.v1 34944227 PMC8697985

[r55] Hoehfurtner T, Wilkinson A, Nagabaskaran G and Burman OH 2021 Does the provision of environmental enrichment affect the behaviour and welfare of captive snakes? Applied Animal Behaviour Science 239: 105324. 10.1016/j.applanim.2021.105324

[r56] Holm S 1979 A simple sequential rejective method procedure. Scandinavian Journal of Statistics 6: 65–70. https://www.jstor.org/stable/4615733

[r57] Hosey G, Melfi V and Ward SJ 2020 Problematic animals in the zoo: the issue of charismatic megafauna. In: Angelici FM and Rossi L (eds) Problematic Wildlife II pp 485–508. Springer: Cham, Switzerland. 10.1007/978-3-030-42335-3_15

[r58] Howard D and Freeman MS 2022 Overlooked and under-Studied: A review of evidence-based enrichment in Varanidae. Journal of Zoological and Botanical Gardens 3: 32–43. 10.3390/jzbg3010003

[r59] Hoy JM, Murray PJ and Tribe A 2010 Thirty years later: Enrichment practices for captive mammals. Zoo Biology 29: 303–316. 10.1002/zoo.20254 19434736

[r60] Janovcová M, Rádlová S, Polák J, Sedláčková K, Peléšková Š, Žampachová B, Frynta D and Landová E 2019 Human attitude toward reptiles: A relationship between fear, disgust, and aesthetic preferences. Animals 9: 238. 10.3390/ani9050238 31091781 PMC6562393

[r61] Jones N, Sherwen SL, Robbins R, McLelland, DJ and Whittaker AL 2022 Welfare assessment tools in zoos: From theory to practice. Veterinary Sciences 9: 170. 10.3390/vetsci9040170 35448668 PMC9025157

[r62] Kagan R, Carter S and Allard S 2015 A universal animal welfare framework for zoos. Journal of Applied Animal Welfare Science 18: S1–S10. 10.1080/10888705.2015.1075830 26440493 PMC4673521

[r63] Kane D, Davis AC and Michaels CJ 2019 Play behaviour by captive tree monitors, *Varanus macraei* and *Varanus prasinus*. Herpetological Bulletin 149: 28–31. 10.33256/hb149.2831

[r64] Kuppert S 2013 Providing enrichment in captive amphibians and reptiles: Is it important to know their communication? Smithsonian Herpetological Information Service 142. 10.13140/2.1.4180.8006

[r65] LaDage LD, Roth TC, Cerjanic AM, Sinervo B and Pravosudov VV 2012 Spatial memory: are lizards really deficient? Biology Letters 8: 939–941. 10.1098/rsbl.2012.0527 22933038 PMC3497115

[r66] Lambert H, Carder G and D’Cruze N 2019 Given the cold shoulder: A review of the scientific literature for evidence of reptile sentience. Animals 9: 821. 10.3390/ani9100821 31627409 PMC6827095

[r67] Learmonth MJ 2020 The matter of non-avian reptile sentience, and why it ‘matters’ to them: A conceptual, ethical and scientific review. Animals 10: 901. 10.3390/ani10050901 32455969 PMC7278454

[r68] Londoño C, Bartolomé A, Carazo P and Font E 2018 Chemosensory enrichment as a simple and effective way to improve the welfare of captive lizards. Ethology 124: 674–683. 10.1111/eth.12800

[r69] Loughman ZJ 2020 Utilization of natural history information in evidence based herpetoculture: A proposed protocol and case study with *Hydrodynastes gigas* (false water cobra). Animals 10: 2021. 10.3390/ani10112021 33153054 PMC7693199

[r70] Malik A 2018 Pain in reptiles: A review for veterinary nurses. Veterinary Nursing Journal 33: 201–211. 10.1080/17415349.2018.1468291

[r71] Maple TL and Perdue BM 2013 Zoo Animal Welfare, *Volume 14*. Springer: Berlin, Germany. 10.1007/978-3-642-35955-2

[r72] Mather JA 2019 Ethics and care: For animals, not just mammals. Animals 9: 1018. 10.3390/ani9121018 31766726 PMC6941085

[r73] Mehrkam LR and Dorey NR 2014 Is preference a predictor of enrichment efficacy in Galapagos tortoises (*Chelonoidis nigra*)? Zoo Biology 33: 275–284. 10.1002/zoo.21151 25065472

[r74] Melfi VA 2009 There are big gaps in our knowledge, and thus approach, to zoo animal welfare: a case for evidence‐based zoo animal management. Zoo Biology 28: 574–588. 10.1002/zoo.20288 19876912

[r75] Melfi VA, Bowkett A, Plowman AB and Pullen K 2005 Do zoo designers know enough about animals? In: Plowman A and Tonge S (Eds.) Innovation or Replication. Proceedings of the 6th International Symposium on Zoo Design pp 119–127. Whitley Wildlife Conservation Trust: Paignton, UK.

[r76] Mellen J and MacPhee MS 2001 Philosophy of environmental enrichment: Past, present, and future. Zoo Biology 20: 211–226. 10.1002/zoo.1021

[r77] Mellor DJ 2016 Updating animal welfare thinking: Moving beyond the ‘Five Freedoms’ towards ‘a Life Worth Living.’ Animals 6: 21. 10.3390/ani6030021 27102171 PMC4810049

[r78] Mellor DJ, Hunt S and Gusset M 2015 Caring for wildlife: The world zoo and aquarium animal welfare strategy. WAZA Executive Office. https://www.waza.org/wp-content/uploads/2019/03/WAZA-Animal-Welfare-Strategy-2015_Landscape.pdf

[r79] Mendyk RW 2018 Challenging folklore reptile husbandry in zoological parks. In: Berger M and Corbett S (Eds.) Zoo Animals: Husbandry, Welfare and Public Interactions pp 256–292. Nova Science Publishers, Inc: Hauppauge, NY, USA.

[r80] Mendyk RW 2022 Recent studies in reptile and amphibian welfare: Some relevant publications for the zoo herpetologist. Herpetological Review 53: 176–180.

[r81] Mendyk RW and Augustine L 2023 Controlled deprivation and enrichment. In: Warwick C, Arena PC and Burghardt GM (Eds.) Health and Welfare of Captive Reptiles, Second Edition pp 323–355. Springer Publishers: London, UK.

[r82] Mendyk RW and Warwick C 2023 Arbitrary husbandry practices and misconceptions. In: Warwick C, Arena PC and Burghardt GM (Eds.) Health and Welfare of Captive Reptiles, Second Edition pp 561–582. Springer Publishers: London, UK.

[r83] Miralles A, Raymond M and Lecointre G 2019 Empathy and compassion toward other species decrease with evolutionary divergence time. Scientific Reports 9: 1–8. 10.1038/s41598-019-56006-9 31862944 PMC6925286

[r84] Newberry RC 1995 Environmental enrichment: Increasing the biological relevance of captive environments. Applied Animal Behaviour Science 44: 229–243. 10.1016/0168-1591(95)00616-z

[r85] Pawar S 2003 Taxonomic chauvinism and the methodologically challenged. Bioscience 53: 861–864. 10.1641/0006-3568(2003)053[0861:tcatmc]2.0.co;2

[r86] Riley LM and Rose PE 2020 Concepts, applications, uses and evaluation of environmental enrichment: Perceptions of zoo professionals. Journal of Zoo and Aquarium Research 8: 18–28. 10.19227/jzar.v8i1.384

[r87] Rivas J and Burghardt GM 2002 Crotalomorphism: A metaphor for understanding anthropomorphism by omission. In: Bekoff M, Allen C, Burghardt GM (Eds.) The Cognitive Animal: Empirical and Theoretical Perspectives on Animal Cognition pp 9–18. MIT Press: Cambridge, MA, USA. 10.7551/mitpress/1885.003.0005

[r88] Rose PE, Brereton JE, Rowden LJ, de Figueiredo, RL and Riley LM 2019 What’s new from the zoo? An analysis of ten years of zoo-themed research output. Palgrave Communications 5: 1–10. 10.1057/s41599-019-0345-3

[r89] Rosier RL and Langkilde T 2011 Does environmental enrichment really matter? A case study using the eastern fence lizard, *Sceloporus undulatus*. Applied Animal Behaviour Science 131: 71–76. 10.1016/j.applanim.2011.01.008

[r90] Shepherdson DJ 2003 Environmental enrichment: Past, present, and future. International Zoo Yearbook 38: 118–124. 10.1111/j.1748-1090.2003.tb02071.x

[r91] Skinner M and Miller N 2020 Aggregation and social interaction in garter snakes (*Thamnophis sirtalis sirtalis*). Behavioral Ecology and Sociobiology 74: 1–13. 10.1007/s00265-020-2827-0

[r92] Spain MS, Fuller G and Allard SM 2020 Effects of habitat modifications on behavioral indicators of welfare for Madagascar giant hognose snakes (*Leioheterodon madagascariensis*). Animal Behavior and Cognition 7: 70–81. 10.26451/abc.07.01.06.2020

[r93] Szabo B, Noble DW and Whiting MJ 2021 Learning in non‐avian reptiles 40 years on: advances and promising new directions. Biological Reviews 96: 331–356. 10.1111/brv.12658 33073470

[r94] Tarou LR and Bashaw MJ 2007 Maximizing the effectiveness of environmental enrichment: Suggestions from the experimental analysis of behavior. Applied Animal Behaviour Science 102: 189–204. 10.1016/j.applanim.2006.05.026

[r95] Therrien CL, Gaster L, Cunningham‐Smith P and Manire CA 2007 Experimental evaluation of environmental enrichment of sea turtles. Zoo Biology 26: 407–416. 10.1002/zoo.20145

[r96] Tuite EK, Moss SA, Phillips CJ and Ward SJ 2022 Why are enrichment practices in zoos difficult to implement effectively? Animals 12: 554. 10.3390/ani12050554 35268123 PMC8908830

[r97] Ward SJ, Sherwen S and Clark FE 2018 Advances in applied zoo animal welfare science. Journal of Applied Animal Welfare Science 21: 23–33. 10.1080/10888705.2018.1513842 30325227

[r98] Warwick C 2014 The morality of the reptile ‘pet’ trade. Journal of Animal Ethics 4: 74–94. 10.5406/j.ctvvnf81.15

[r99] Warwick C, Arena P, Lindley S, Jessop M and Steedman C 2013 Assessing reptile welfare using behavioural criteria. *In* Practice 35: 123–131. 10.1136/inp.f1197

[r100] Warwick C, Arena P and Steedman C 2019 Spatial considerations for captive snakes. Journal of Veterinary Behavior 30: 37–48. 10.1016/j.jveb.2018.12.006

[r101] Warwick C, Frye FL and Murphy JB 1994 Health and Welfare of Captive Reptiles. Chapman & Hall/Kluwer: London, UK; New York, NJ, USA.

[r102] Warwick C, Grant R, Steedman C, Howell TJ, Arena PC, Lambiris AJ, Nash A, Jessop M, Pilny A, Amarello M, Gorzula S, Spain M, Walton A, Nicholas E, Mancera K, Whitehead M, Martínez-Silvestre A, Cadenas V, Whittaker A and Wilson A 2021 Getting it straight: Accommodating rectilinear behavior in captive snakes—A review of recommendations and their evidence base. Animals 11: 1459. 10.3390/ani11051459 34069685 PMC8160691

[r103] Warwick C, Jessop M, Arena P, Pliny A, Nicholas E and Lambiris A 2017 Future of keeping pet reptiles and amphibians: Animal welfare and public health perspective. Veterinary Record 181: 454–455. 10.1136/vr.j4640 29074796

[r104] Warwick C, Steedman C, Jessop M, Arena P, Pilny A and Nicholas E 2018 Exotic pet suitability: Understanding some problems and using a labelling system to aid animal welfare, environment, and consumer protection. Journal of Veterinary Behavior 26: 17–26. 10.1016/j.jveb.2018.03.015

[r105] Watters JV 2009 Toward a predictive theory for environmental enrichment. Zoo Biology 28: 609–622. 10.1002/zoo.20284 19830747

[r106] Whitehead ML 2018 Factors contributing to poor welfare of pet reptiles. Testudo 8: 47–61.

[r107] Whitham JC and Wielebnowski N 2013 New directions for zoo animal welfare science. Applied Animal Behaviour Science 147: 247–260. 10.1016/j.applanim.2013.02.004

[r108] Whittaker AL, Golder-Dewar B, Triggs JL, Sherwen SL and McLelland DJ 2021 Identification of animal-based welfare indicators in captive reptiles: A delphi consultation survey. Animals 11: 2010. 10.3390/ani11072010 34359138 PMC8300299

[r109] Wilkins AM, McCrae LS and McBride EA 2015 Factors affecting the human attribution of emotions toward animals. Anthrozoös 28: 357–369. 10.1080/08927936.2015.1052270

[r110] Williams E, Chadwick CL, Yon L and Asher L 2018 A review of current indicators of welfare in captive elephants (*Loxodonta africana* and *Elephas maximus*). Animal Welfare 27: 235–249. 10.7120/09627286.27.3.235

[r111] Williams J and Beck D 2021 Stress, anxiety, fear and frustration in different reptile species: How to reduce these negative emotional states during veterinary procedures. Veterinary Nursing Journal 36: 213–216. 10.1080/17415349.2021.1936322

[r112] Yeates J 2016 Quality of life and animal behaviour. Applied Animal Behaviour Science 181: 19–26. 10.1016/j.applanim.2016.04.018

[r113] Yeates JW and Main DC 2008 Assessment of positive welfare: A review. The Veterinary Journal 175: 293–300. 10.1016/j.tvjl.2007.05.009 17613265

[r114] Yon L, Williams E, Harvey ND and Asher L 2019 Development of a behavioural welfare assessment tool for routine use with captive elephants*. PLoS ONE* 14: e0210783. 10.1371/journal.pone.0210783 PMC6364905 30726232

[r115] Young RJ 2003 Environmental Enrichment for Captive Animals. Blackwell Science: London, UK. 10.1002/9780470751046

