# Supplementary material for: Environmental enrichment for reptiles in European zoos: Current status and perspectives
Source: Anim Welf. 2023 Jul 3;32:e48. doi: 10.1017/awf.2023.43 (PMC10936270; doi:10.1017/awf.2023.43)
Supplement: Supplementary file 1 [file S096272862300043Xsup001.pdf]

Table S1 Frequencies of each specific enrichment technique reported in the follow-up survey

Enrichment technique	*Percentage*	Specific enrichment technique	*Percentage*
		**Non-venomous snakes**	
Structural/Thermal complexity	24.2%	Different substrates	33.3%
		Climbing structures	23.1%
		Structural complexity	15.4%
		Terrestrial and aquatic environments	15.4%
		Others	12.8%
Enrichment objects	15.5%	Natural objects	68%
		Others	32%
Cohabit	12.4%	Conspecific cohabit	60%
		Others	40%
Olfactory enrichment	13.7%	Scents from other individuals	36.3%
		Scent trails	31.2%
		Others	32%
Varying feeding presentation/patterns	9.3%	Hiding food	46.7 %
		Food chase	33.3%
		Others	20%
Varying furniture	9.3%		
		**Venomous snakes**	
Structural/Thermal complexity	26.1%	Climbing structures	33.3%
		Different substrates	16.7%
		Terrestrial and aquatic environments	16.7%
		Mimicking natural habitat	16.7%
		Others	16.7%
Cohabit	17.4%	Conspecific cohabit	50%
		Unspecified cohabit	50%
Olfactory enrichment	17.4%	Scent trails	50%
		Scents from other individuals	25%
		Others	25%
Varying feeding presentation/patterns	13%	Hiding food	66.7%
		Others	33.3%
Enrichment objects	13%	Natural objects	66.7 %

		Others	33.3%
		**Turtles**	
Cohabit	20%	Heterospecific cohabit	41.7%
		Conspecific cohabit	41.7%
		Unspecified cohabit	16.7%
Enrichment objects	14.2%	Natural objects	70.6%
		Others	29.4%
Different food items	13.3%		
Structural/Thermal complexity	12.5%	Structural complexity	26.7%
		Different substrates	26.7%
		Others	46.7%
Varying feeding presentation/patterns	9.2%	Varying food presentation	27.3%
		Hanging food	27.3%
		Others	45.5%
Varying furniture	9.2%		
		**Tortoises**	
Cohabit	16%	Conspecific cohabit	41.9%
		Heterospecific cohabit	29%
		Unspecified cohabit	29%
Enrichment objects	14.4%	Natural objects	57.1%
		Toys	17.9%
		Others	25%
Varying feeding presentation/patterns	12.9%	Hanging food	40%
		Hiding food	20%
		Varying food presentation	12%
		Varying feeding patterns	12%
		Others	16%
Structural/Thermal complexity	12.4%	Different substrates	45.8%
		Terrestrial and aquatic environments	16.7%
		Others	37.5%
Different food items	12.4%		

		**Crocodilians**	
Associative learning	20%	Behavioural conditioning for eating/cleaning	33.3%
		Behavioural conditioning for medical procedures	28.6%
		Target training	28.6%
		Others	9.5%
Structural/Thermal complexity	18.1%	Different substrates	26.3%
		Nesting area	15.8%
		Terrestrial and aquatic environments	15.8%
		Structural complexity	15.8%
		Others	26.3%
Cohabit	14.3%	Conspecific cohabit	53.3%
		Heterospecific cohabit	40%
		Unspecified cohabit	6.7%
Enrichment objects	14.3%	Natural objects	86.7%
		Toys	13.3%
Different food items	10.5%		
		**Monitor lizards**	
Structural/Thermal complexity	17%	Structural complexity	31.9%
		Different substrates	22.8%
		Climbing structures	18.2%
		Terrestrial and aquatic environments	13.7%
		Others	13.4%
Varying feeding presentation/patterns	16.3%	Hiding food	38.1%
		Varying feeding patterns	19%
		Hanging food	14.3%
		Scatter feeding	14.3%
		Others	14.3%
Associative learning	13.2%	Target training	41.2%
		Behavioural conditioning for medical procedures	29.4%
		Behavioural conditioning for eating/cleaning	23.5%
		Others	5.9%

Enrichment objects	13.2%	Natural objects	64.7%
		Man-made objects	17.6%
		Others	17.6%
Different food items	8.5%		
		**Non-monitor lizards**	
Structural/Thermal complexity	18.3%	Different substrates	29.2%
		Climbing structures	25%
		Structural complexity	16.7%
		Terrestrial and aquatic environments	16.7%
		Others	12.5%
Cohabit	13%	Conspecific cohabit	52.9%
		Heterospecific cohabit	29.4%
		Unspecified cohabit	17.6%
Enrichment objects	12.2%	Natural objects	75%
		Others	25%
Varying feeding presentation/patterns	11.5%	Hiding food	33.3%
		Hanging food	20%
		Others	46.7%
Different food items	9.2%		
Olfactory enrichment	9.2%	Fragrant substances	50%
o		Scent trails	25%
		Others	25%

Table S2. Percentage distribution of the different types of institutions identified by respondents in the study[1]

**Type of facility**	*Percentage*
Zoo or animal park	89%
Aquarium	12%
Research and/or conservation centre	11%
Herpetarium	10%
Petting zoo or petting farm	2%
Safari Park	2%
Animal theme park	2%
Private zoological collection	2%
Wildlife reserve	1%
Itinerate zoological exhibition	1%
Other	2%

[1] Percentages calculated out of total of zoos that participated in the main survey (N = 121). As each institution could chose more than one label to best identify their facilities, the sum of percentages exceeds 100.

Table S3. Percentage distribution of the different ranges regarding the total number of animal species living in the participants' zoological institutions[1]

**Number of species**	*Percentage*
Fewer than 100	26%
100–200	38%
200–300	8%
300–400	12%
400–500	5%
500–600	1%
More than 600	10%
N/A	1%

[1] Percentages calculated out of total of zoos that participated in the main survey (N = 121).

Table S4. Percentage distribution of the different ranges regarding the total number of reptiles specimens in the participants' zoological institutions[1]

**Number of animals**	*Percentage*
Fewer than 10	14%
10–20	10%
20–30	8%
30–40	10%
40–50	8%
More than 50	18%
More than 100	16%
More than 200	11%
N/A	4%

[1] Percentages calculated out of total of zoos that participated in the main survey and used environmental enrichment (N = 118).

Table S5. Number of zoos contacted, responses received and percentage of response from each European country.

**Country**	**Zoos contacted**	**Number of responses**	*Percentage*
UK	44	20	45
Spain	38	19	50
Germany	71	16	23
France	57	12	21
Switzerland	13	8	62
Czech Republic	20	5	25
Netherlands	12	5	42
Italy	16	4	25
Sweden	13	4	31
Austria	10	3	30
Denmark	6	2	33
Belgium	7	2	29
Ireland	6	2	33
Romania	8	2	25
Belarus	1	1	100
Bulgaria	2	1	50
Finland	4	1	25
Hungary	7	1	14
Lithuania	1	1	100
Luxemburg	1	1	100
Macedonia	1	1	100
Norway	6	1	17
Poland	13	1	8
Slovakia	3	1	33
Turkey	2	1	50
Russia	8	1	13
Ukraine	4	1	25
Croatia	1	0	0
Estonia	1	0	0
Greece	1	0	0
Portugal	6	0	0
Serbia	1	0	0
**Total**	**384**	**121**	
